# The genome sequence of the Fig-leaf Skeletoniser, *Choreutis nemorana *(Hübner, [1799])

**DOI:** 10.12688/wellcomeopenres.21624.1

**Published:** 2024-05-15

**Authors:** David C. Lees, Natural History Museum Genome Acquisition Lab, Darwin Tree of Life Barcoding collective, Wellcome Sanger Institute Tree of Life Management, Samples and Laboratory team, Wellcome Sanger Institute Scientific Operations: Sequencing Operations, Wellcome Sanger Institute Tree of Life Core Informatics team, Tree of Life Core Informatics collective, Darwin Tree of Life Consortium

**Affiliations:** 1 Natural History Museum, London, England, UK

**Keywords:** Choreutis nemorana, Fig-leaf Skeletoniser, genome sequence, chromosomal, Lepidoptera

## Abstract

We present a genome assembly from an individual male *Choreutis nemorana* (the Fig-leaf Skeletoniser; Arthropoda; Insecta; Lepidoptera; Choreutidae). The genome sequence is 300.2 megabases in span. Most of the assembly is scaffolded into 31 chromosomal pseudomolecules, including the Z sex chromosome. The mitochondrial genome has also been assembled and is 15.52 kilobases in length. Gene annotation of this assembly on Ensembl identified 15,351 protein coding genes.

## Species taxonomy

Eukaryota; Opisthokonta; Metazoa; Eumetazoa; Bilateria; Protostomia; Ecdysozoa; Panarthropoda; Arthropoda; Mandibulata; Pancrustacea; Hexapoda; Insecta; Dicondylia; Pterygota; Neoptera; Endopterygota; Amphiesmenoptera; Lepidoptera; Glossata; Neolepidoptera; Heteroneura; Ditrysia; Apoditrysia;Choreutoidea; Choreutidae; Choreutinae; *Choreutis*; *Choreutis nemorana* (Hübner, [1799]) (NCBI:txid1209621).

## Background

*Choreutis nemorana* (Hübner, [1799]) also known as the Fig-leaf Skeletoniser ( Sterling *et al.*, 2023), is a moth in family Choreutidae with a wingspan of 16–20 mm (8–10 mm forewing length; Stojanović *et al.*, 2020; Sterling *et al.*, 2023). Its forewing has a rounded costa, with apex slightly projecting and sinuate termen accentuated by its white fringe, and its background colour is usually orange-brown (or sometimes dark greyish) with four irregular thin white crosslines (the last submarginal) and the subterminal area is usually darker orange. Like other choreutids, it holds its wings upwards and somewhat furled when active and runs in a jerky motion on the leaf surface. The legs are clothed in white scales as if covered in fungus, perhaps advantageous for winter hibernation. *C. nemorana* differs from other Choreutidae (at least among European species) in its orange wing colouration which distinguishes it from the otherwise similar *C. pariana* (Clerck, 1759).

*C. nemorana* is a native of Europe, first adventive to the UK in 2014 (Kensington, Gardens, London) ( De Prins & De Prins, 2014) and is now spreading rapidly, recorded in Kent, Hertfordshire, East Anglia, Hampshire, Dorset and the Isle of Wight. The first reported breeding population in the UK was found in Kensington Gardens, London ( De Prins & De Prins, 2014). The species is particularly prevalent in the Mediterranean area where its foodplant naturally occurs, also occurring in north-western Africa ( De Prins *et al*., 2014). Elsewhere, the species is widespread from the Macaronesian islands, through Europe to western Asia including Asia Minor, Caucasus, Turkmenistan and Uzbekistan (west of the Caspian Sea) but its distribution does not extend as far north as Scandinavia ( GBIF Secretariat, 2024); however, there are two records from China ( De Prins *et al.*, 2014). There are strong signs that the species is spreading rather rapidly north and west ( Stojanović *et al*., 2020), being found first in Belgium in 2009 ( De Prins *et al.*, 2014); Lepiforum (2024) gives a summary by country of its current distribution in Europe.

*C. nemorana* feeds exclusively as a larva on *Ficus* (Moraceae) and is apparently monophagous on *F. carica* L., being considered a minor pest of figs ( Alford, 2007); it can occasionally cause extensive damage to *F. carica* plants in the London area and in Europe ( Ellis, 2024). Its spread may have been exacerbated by a combination of the plant trade ( De Prins *et al.*, 2014) and global warming ( Stojanović *et al.*, 2020).

The greenish yellow larva, with each thoracic and abdominal segments bearing about eight black shiny pinacula, has been found in the UK from late May to early November, feeding underneath a web on the upper surface of leaves of *Ficus carica* where its eggs are laid ( De Prins *et al.*, 2014) and which the larvae skeletonise ( Sterling *et al.,* 2023). The larvae are very active if disturbed and may drop to the ground ( De Prins *et al.*, 2014). The brownish black pupa is found in a whitish silken fold at the leaf margin. The diurnal adult which appears in at least two broods between July to October ( Norfolk Moths, 2024); the species is bivoltine in Europe ( De Prins *et al.*, 2014). The moth hibernates as an adult, including in thatch and among dead leaves in hedgerows ( De Prins *et al.*, 2014). The adult nectars on a wide range of flowers.

The parasitoids of *C. nemorana* include the ichneumonid *Triclistus anthophilae* Aeschlimann, 1983 (Ichneumonidae: Metopiinae) (a parasitoid on other choreutids), *and Stenomesius rufescens* (Retzius, 1783) (Eulophidae) ( Shaw, 2017; q.v. for a full list of 13 species also comprising the families Braconidae, Pteromalidae, Bethylidae and Tachinidae).

*C. nemorana* is a species that is somewhat isolated from other Choreutidae, falling over 7.1% pairwise divergent in COI-5P to *C. pariana* and *C. aegyptiaca* (Zeller, 1867). In the phylogenetic analysis of Rota *et al.* (2016), *C. nemorana* is recovered as sister to a distal clade among *Choreutis* that includes the Oriental *C. euclista* (Meyrick, 1918), the Palaearctic *C. aegyptiaca*, *C. pariana, C. diana* (Hübner, 1822) and eight other species, with an estimated divergence of around 17–20 Ma. The mitogenome from the genome assembly (OX438626.1) represents for COI-5P the most common haplotype from Europe on BOLD (28/03/2024) and belongs to the single BIN cluster BOLD:AAL6537. The nearest neighbour on BOLD is *Choreutis* sp. ‘JDP1’ of Rota *et al.*, 2016 (KT956504, BOLD:ADR7873). The genome will be helpful, e.g. in further molecular taxonomic work on *Choreutis*.

## Genome sequence report

**Figure 1.**
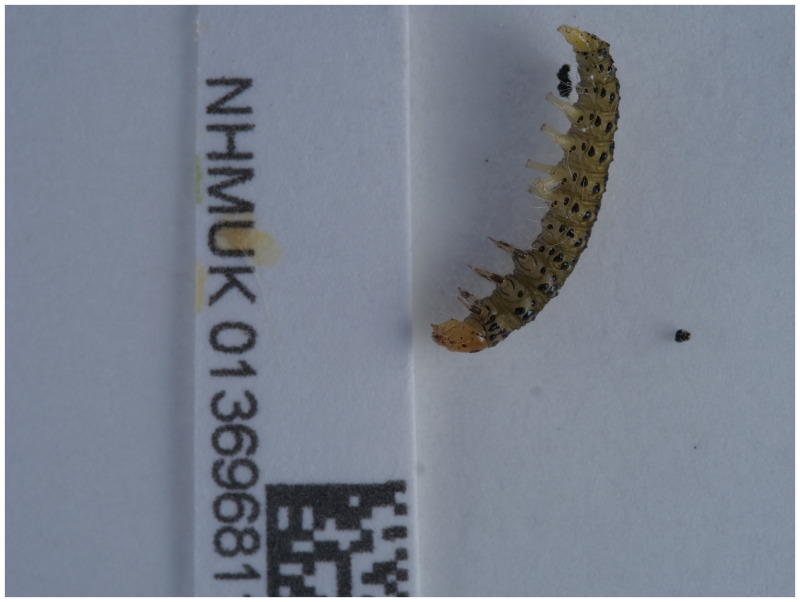
Photograph of the *Choreutis nemorana* (ilChoNemo1) specimen used for genome sequencing.

The genome was sequenced from a male *Choreutis nemorana* ( Figure 1) collected from Kensington Gardens, London, UK (51.51, –0.17). A total of 77-fold coverage in Pacific Biosciences single-molecule HiFi long reads was generated. Primary assembly contigs were scaffolded with chromosome conformation Hi-C data. Manual assembly curation corrected 6 missing joins or mis-joins, reducing the scaffold number by 1.03%, and increasing the scaffold N50 by 0.23%.

**Figure 2.**
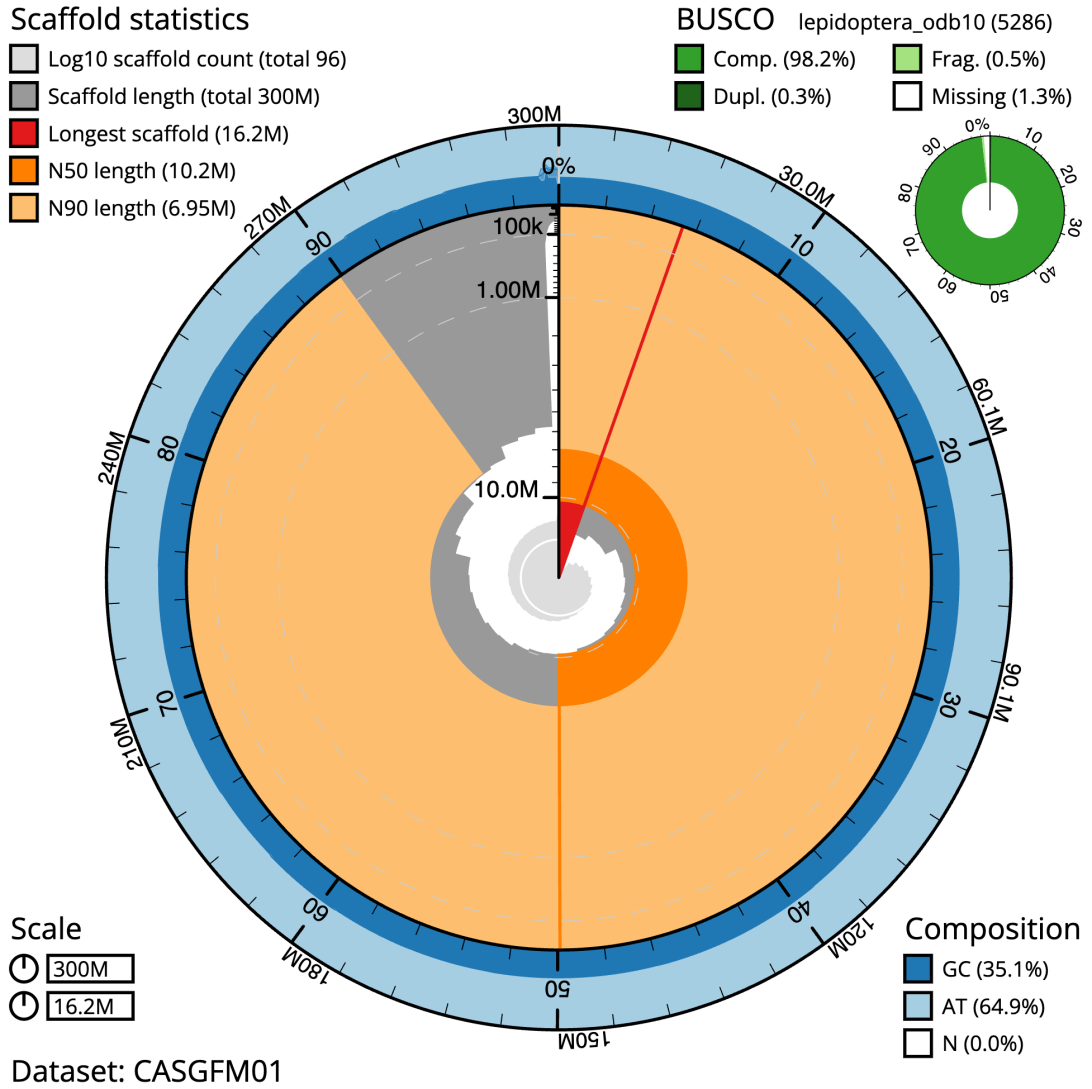
Genome assembly of *Choreutis nemorana*, ilChoNemo1.1: metrics. The BlobToolKit snail plot shows N50 metrics and BUSCO gene completeness. The main plot is divided into 1,000 size-ordered bins around the circumference with each bin representing 0.1% of the 300,258,981 bp assembly. The distribution of scaffold lengths is shown in dark grey with the plot radius scaled to the longest scaffold present in the assembly (16,217,284 bp, shown in red). Orange and pale-orange arcs show the N50 and N90 scaffold lengths (10,167,060 and 6,945,856 bp), respectively. The pale grey spiral shows the cumulative scaffold count on a log scale with white scale lines showing successive orders of magnitude. The blue and pale-blue area around the outside of the plot shows the distribution of GC, AT and N percentages in the same bins as the inner plot. A summary of complete, fragmented, duplicated and missing BUSCO genes in the lepidoptera_odb10 set is shown in the top right. An interactive version of this figure is available at https://blobtoolkit.genomehubs.org/view/CASGFM01/dataset/CASGFM01/snail.

**Figure 3.**
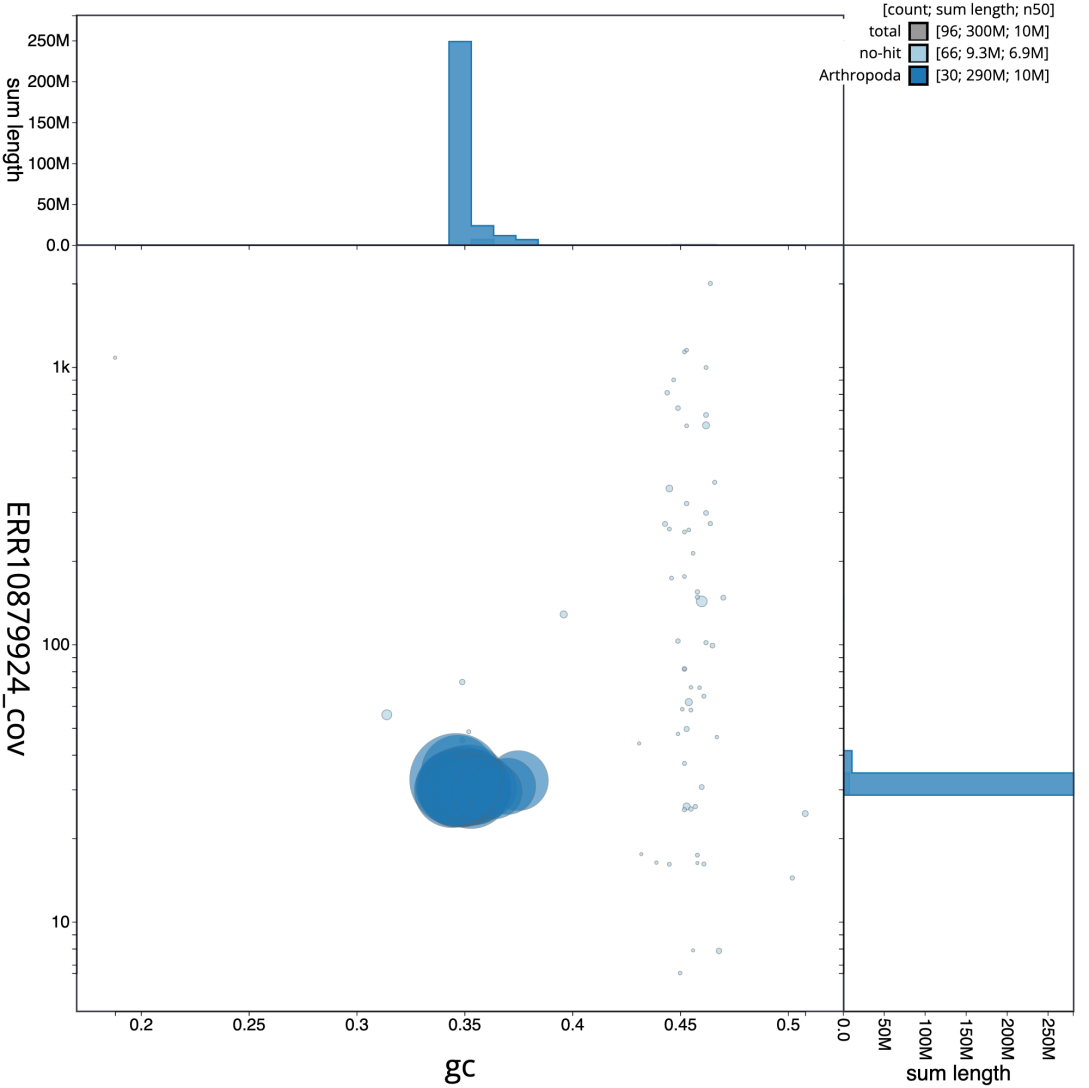
Genome assembly of *Choreutis nemorana*, ilChoNemo1.1: BlobToolKit GC-coverage plot. Sequences are coloured by phylum. Circles are sized in proportion to sequence length. Histograms show the distribution of sequence length sum along each axis. An interactive version of this figure is available at https://blobtoolkit.genomehubs.org/view/CASGFM01/dataset/CASGFM01/blob.

**Figure 4.**
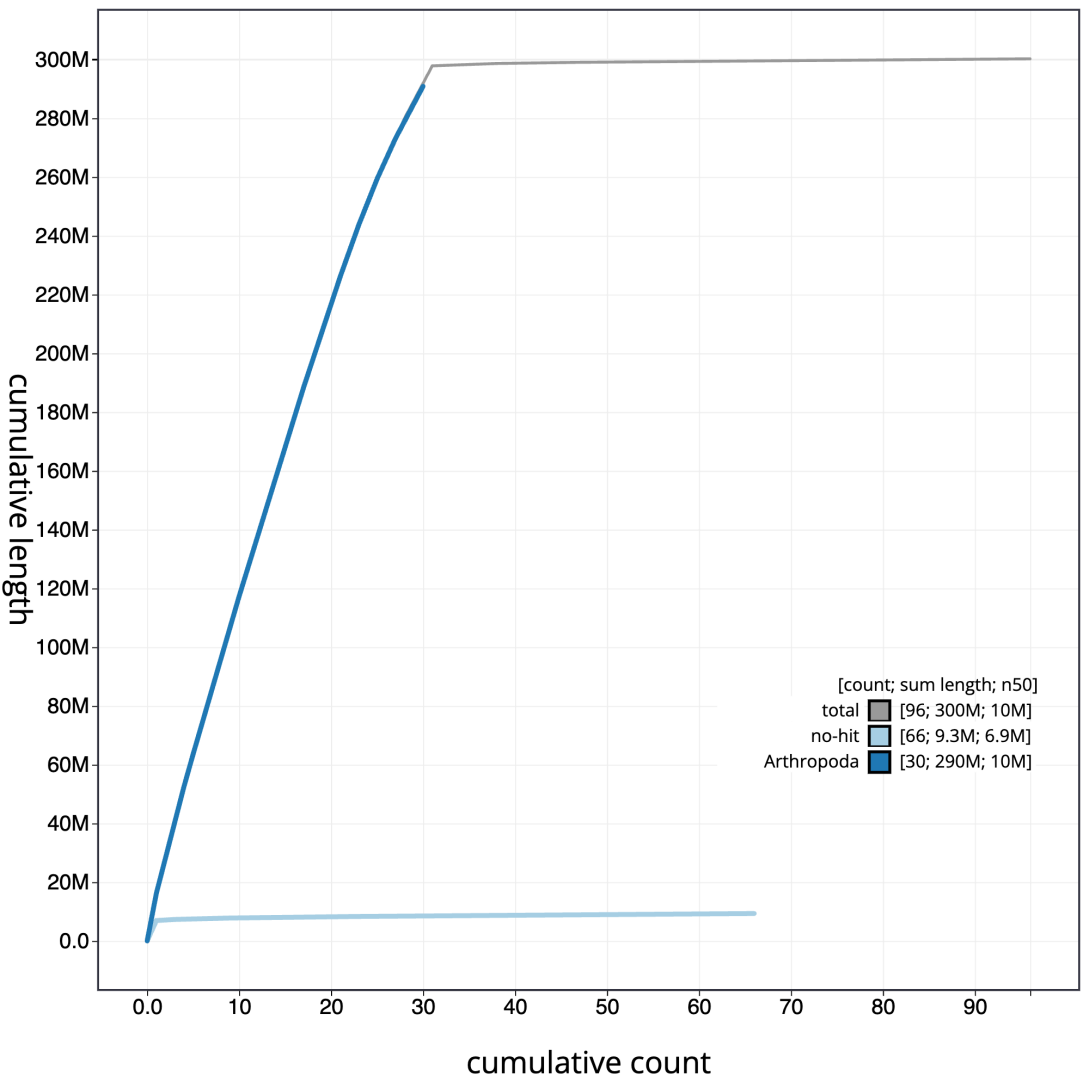
Genome assembly of *Choreutis nemorana*, ilChoNemo1.1: BlobToolKit cumulative sequence plot. The grey line shows cumulative length for all sequences. Coloured lines show cumulative lengths of sequences assigned to each phylum using the buscogenes taxrule. An interactive version of this figure is available at https://blobtoolkit.genomehubs.org/view/CASGFM01/dataset/CASGFM01/cumulative.

**Figure 5.**
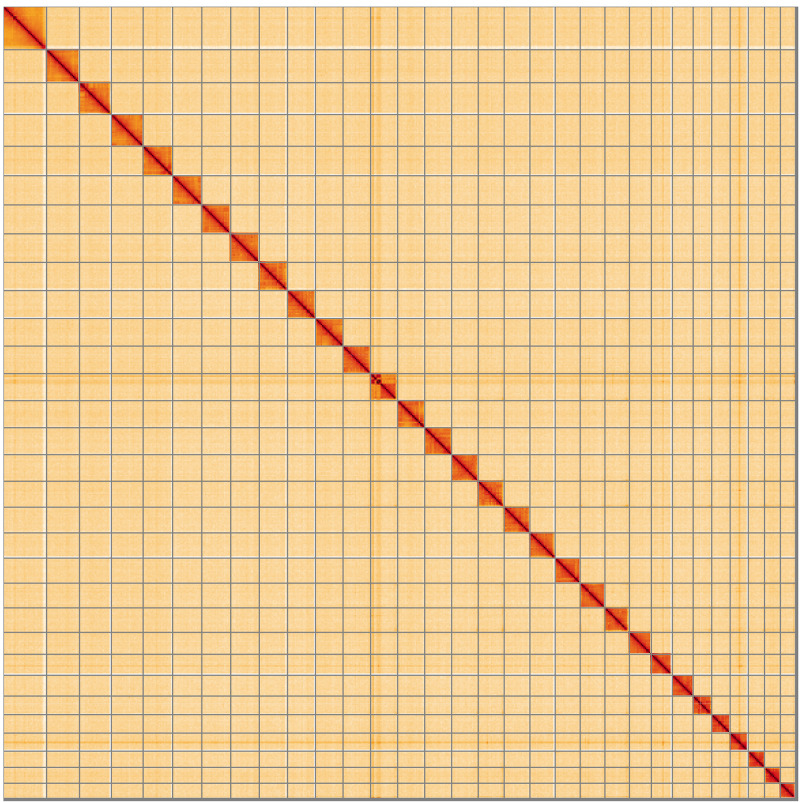
Genome assembly of *Choreutis nemorana*, ilChoNemo1.1: Hi-C contact map of the ilChoNemo1.1 assembly, visualised using HiGlass. Chromosomes are shown in order of size from left to right and top to bottom. An interactive version of this figure may be viewed at https://genome-note-higlass.tol.sanger.ac.uk/l/?d=d4lxwUO7RY--rBe8qZtHdQ.

The final assembly has a total length of 300.2 Mb in 95 sequence scaffolds with a scaffold N50 of 10.2 Mb ( Table 1). The snail plot in Figure 2 provides a summary of the assembly statistics, while the distribution of assembly scaffolds on GC proportion and coverage is shown in Figure 3. The cumulative assembly plot in Figure 4 shows curves for subsets of scaffolds assigned to different phyla. Most (99.25%) of the assembly sequence was assigned to 31 chromosomal-level scaffolds, representing 30 autosomes and the Z sex chromosome. Chromosome-scale scaffolds confirmed by the Hi-C data are named in order of size ( Figure 5; Table 2). While not fully phased, the assembly deposited is of one haplotype. Contigs corresponding to the second haplotype have also been deposited. The mitochondrial genome was also assembled and can be found as a contig within the multifasta file of the genome submission.

**Table 1.** Genome data for *Choreutis nemorana*, ilChoNemo1.1.

Project accession data		
Assembly identifier	ilChoNemo1.1	
Species	*Choreutis nemorana*	
Specimen	ilChoNemo1	
NCBI taxonomy ID	1209621	
BioProject	PRJEB59772	
BioSample ID	SAMEA111458725	
Isolate information	ilChoNemo1, larva: whole organism (genome and Hi-C sequencing)	
Assembly metrics *		*Benchmark*
Consensus quality (QV)	67.6	*≥ 50*
*k*-mer completeness	100.0%	*≥ 95%*
BUSCO **	C:98.2%[S:97.9%,D:0.3%],F:0.5%,M:1.3%,n:5,286	*C ≥ 95%*
Percentage of assembly mapped to chromosomes	99.25%	*≥ 95%*
Sex chromosomes	Z	*localised homologous pairs*
Organelles	Mitochondrial genome: 15.52 kb	*complete single alleles*
Raw data accessions		
PacificBiosciences SEQUEL II	ERR10879924	
Hi-C Illumina	ERR10890718	
Genome assembly		
Assembly accession	GCA_949316135.1	
*Accession of alternate haplotype*	GCA_949316125.1	
Span (Mb)	300.2	
Number of contigs	128	
Contig N50 length (Mb)	7.0	
Number of scaffolds	95	
Scaffold N50 length (Mb)	10.2	
Longest scaffold (Mb)	16.22	
Genome annotation		
Number of protein-coding genes	15,351	
Number of gene transcripts	15,541	

* Assembly metric benchmarks are adapted from column VGP-2020 of “Table 1: Proposed standards and metrics for defining genome assembly quality” from Rhie *et al.* (2021). ** BUSCO scores based on the lepidoptera_odb10 BUSCO set using version 5.3.2. C = complete [S = single copy, D = duplicated], F = fragmented, M = missing, n = number of orthologues in comparison. A full set of BUSCO scores is available at https://blobtoolkit.genomehubs.org/view/CASGFM01/dataset/CASGFM01/busco.

**Table 2.** Chromosomal pseudomolecules in the genome assembly of *Choreutis nemorana*, ilChoNemo1.

INSDC accession	Chromosome	Length (Mb)	GC%
OX438596.1	1	12.39	35.0
OX438597.1	2	11.97	35.5
OX438598.1	3	11.94	35.0
OX438599.1	4	11.08	35.5
OX438600.1	5	10.99	34.5
OX438601.1	6	10.88	34.5
OX438602.1	7	10.74	35.0
OX438603.1	8	10.62	34.5
OX438604.1	9	10.49	34.5
OX438605.1	10	10.38	35.0
OX438606.1	11	10.37	35.0
OX438607.1	12	10.17	34.5
OX438608.1	13	10.17	35.0
OX438609.1	14	10.14	34.5
OX438610.1	15	10.0	34.5
OX438611.1	16	9.75	35.5
OX438612.1	17	9.68	34.5
OX438613.1	18	9.5	35.0
OX438614.1	19	9.41	35.0
OX438615.1	20	9.3	34.5
OX438616.1	21	9.22	35.0
OX438617.1	22	8.25	35.0
OX438618.1	23	7.87	36.0
OX438619.1	24	7.83	35.5
OX438620.1	25	6.98	34.5
OX438621.1	26	6.95	35.5
OX438622.1	27	6.79	37.5
OX438623.1	28	6.12	35.5
OX438624.1	29	5.96	37.0
OX438625.1	30	5.69	36.5
OX438595.1	Z	16.22	34.5
OX438626.1	MT	0.02	19.0

The estimated Quality Value (QV) of the final assembly is 67.6 with *k*-mer completeness of 100.0%, and the assembly has a BUSCO v5.3.2 completeness of 98.2% (single = 97.9%, duplicated = 0.3%), using the lepidoptera_odb10 reference set ( *n* = 5,286).

Metadata for specimens, barcode results, spectra estimates, sequencing runs, contaminants and pre-curation assembly statistics are given at https://links.tol.sanger.ac.uk/species/1209621.

## Genome annotation report

The *Choreutis nemorana* genome assembly (GCA_949316135.1) was annotated at the European Bioinformatics Institute (EBI) on Ensembl Rapid Release. The resulting annotation includes 15,541 transcribed mRNAs from 15,351 protein-coding genes ( Table 1; https://rapid.ensembl.org/Choreutis_nemorana_GCA_949316135.1/Info/Index).

## Methods

### Sample acquisition and nucleic acid extraction

A *Choreutis nemorana* caterpillar (specimen ID NHMUK013696811, ToLID ilChoNemo1) was collected by hand from Kensington Gardens, London, UK (latitude 51.51, longitude –0.17) on 2021-07-13. The specimen was collected and identified by David Lees (Natural History Museum) and preserved by dry-freezing at –80 °C.

The workflow for high molecular weight (HMW) DNA extraction at the Wellcome Sanger Institute (WSI) includes a sequence of core procedures: sample preparation; sample homogenisation, DNA extraction, fragmentation, and clean-up. The sample was prepared for DNA extraction at the WSI Tree of Life Core Laboratory. The ilChoNemo1 sample was weighed and dissected on dry ice ( Jay *et al.*, 2023). Tissue from the whole organism was homogenised using a PowerMasher II tissue disruptor ( Denton *et al.*, 2023a).

HMW DNA was extracted in the WSI Scientific Operations core using the Automated MagAttract v2 protocol ( Oatley *et al.*, 2023). The DNA was sheared into an average fragment size of 12–20 kb in a Megaruptor 3 system with speed setting 31 ( Bates *et al.*, 2023). Sheared DNA was purified by solid-phase reversible immobilisation ( Strickland *et al.*, 2023): in brief, the method employs a 1.8X ratio of AMPure PB beads to sample to eliminate shorter fragments and concentrate the DNA. The concentration of the sheared and purified DNA was assessed using a Nanodrop spectrophotometer and Qubit Fluorometer and Qubit dsDNA High Sensitivity Assay kit. Fragment size distribution was evaluated by running the sample on the FemtoPulse system.

Protocols developed by the WSI Tree of Life laboratory are publicly available on protocols.io ( Denton *et al.*, 2023b).

### Sequencing

Pacific Biosciences HiFi circular consensus DNA sequencing libraries were constructed according to the manufacturers’ instructions. DNA sequencing was performed by the Scientific Operations core at the WSI on a Pacific Biosciences SEQUEL II instrument. Hi-C data were also generated from remaining tissue of ilChoNemo1 using the Arima2 kit and sequenced on the Illumina NovaSeq 6000 instrument.

### Genome assembly, curation and evaluation

Assembly was carried out with Hifiasm ( Cheng *et al.*, 2021) and haplotypic duplication was identified and removed with purge_dups ( Guan *et al.*, 2020). The assembly was then scaffolded with Hi-C data ( Rao *et al.*, 2014) using YaHS ( Zhou *et al.*, 2023). The assembly was checked for contamination and corrected as described previously ( Howe *et al.*, 2021). Manual curation was performed using HiGlass ( Kerpedjiev *et al.*, 2018) and PretextView ( Harry, 2022). The mitochondrial genome was assembled using MitoHiFi ( Uliano-Silva *et al.*, 2023), which runs MitoFinder ( Allio *et al.*, 2020) or MITOS ( Bernt *et al.*, 2013) and uses these annotations to select the final mitochondrial contig and to ensure the general quality of the sequence.

A Hi-C map for the final assembly was produced using bwa-mem2 ( Vasimuddin *et al.*, 2019) in the Cooler file format ( Abdennur & Mirny, 2020). To assess the assembly metrics, the *k*-mer completeness and QV consensus quality values were calculated in Merqury ( Rhie *et al.*, 2020). This work was done using Nextflow ( Di Tommaso *et al.*, 2017) DSL2 pipelines “sanger-tol/readmapping” ( Surana *et al.*, 2023a) and “sanger-tol/genomenote” ( Surana *et al.*, 2023b). The genome was analysed within the BlobToolKit environment ( Challis *et al.*, 2020) and BUSCO scores ( Manni *et al.*, 2021; Simão *et al.*, 2015) were calculated.

Table 3 contains a list of relevant software tool versions and sources.

**Table 3.** Software tools: versions and sources.

Software tool	Version	Source
BlobToolKit	4.1.7	https://github.com/blobtoolkit/blobtoolkit
BUSCO	5.3.2	https://gitlab.com/ezlab/busco
Hifiasm	0.16.1-r375	https://github.com/chhylp123/hifiasm
HiGlass	1.11.6	https://github.com/higlass/higlass
Merqury	MerquryFK	https://github.com/thegenemyers/MERQURY.FK
MitoHiFi	2	https://github.com/marcelauliano/MitoHiFi
PretextView	0.2	https://github.com/wtsi-hpag/PretextView
purge_dups	1.2.3	https://github.com/dfguan/purge_dups
sanger-tol/genomenote	v1.0	https://github.com/sanger-tol/genomenote
sanger-tol/readmapping	1.1.0	https://github.com/sanger-tol/readmapping/tree/1.1.0
YaHS	1.2a	https://github.com/c-zhou/yahs

### Genome annotation

The BRAKER2 pipeline ( Brůna *et al.*, 2021) was used in the default protein mode to generate annotation for the *Choreutis nemorana* assembly (GCA_949316135.1) in Ensembl Rapid Release at the EBI.

### Wellcome Sanger Institute – Legal and Governance

The materials that have contributed to this genome note have been supplied by a Darwin Tree of Life Partner. The submission of materials by a Darwin Tree of Life Partner is subject to the **‘Darwin Tree of Life Project Sampling Code of Practice’**, which can be found in full on the Darwin Tree of Life website here. By agreeing with and signing up to the Sampling Code of Practice, the Darwin Tree of Life Partner agrees they will meet the legal and ethical requirements and standards set out within this document in respect of all samples acquired for, and supplied to, the Darwin Tree of Life Project.

Further, the Wellcome Sanger Institute employs a process whereby due diligence is carried out proportionate to the nature of the materials themselves, and the circumstances under which they have been/are to be collected and provided for use. The purpose of this is to address and mitigate any potential legal and/or ethical implications of receipt and use of the materials as part of the research project, and to ensure that in doing so we align with best practice wherever possible. The overarching areas of consideration are:

•      Ethical review of provenance and sourcing of the material

•      Legality of collection, transfer and use (national and international)

Each transfer of samples is further undertaken according to a Research Collaboration Agreement or Material Transfer Agreement entered into by the Darwin Tree of Life Partner, Genome Research Limited (operating as the Wellcome Sanger Institute), and in some circumstances other Darwin Tree of Life collaborators.

## Data Availability

European Nucleotide Archive: *Choreutis nemorana*. Accession number PRJEB59772; https://identifiers.org/ena.embl/PRJEB59772 ( Wellcome Sanger Institute, 2023). The genome sequence is released openly for reuse. The *Choreutis nemorana* genome sequencing initiative is part of the Darwin Tree of Life (DToL) project. All raw sequence data and the assembly have been deposited in INSDC databases. Raw data and assembly accession identifiers are reported in Table 1.

## Author information

Members of the Natural History Museum Genome Acquisition Lab are listed here: https://doi.org/10.5281/zenodo.7139035.

Members of the Darwin Tree of Life Barcoding collective are listed here: https://doi.org/10.5281/zenodo.4893703.

Members of the Wellcome Sanger Institute Tree of Life Management, Samples and Laboratory team are listed here: https://doi.org/10.5281/zenodo.10066175.

Members of Wellcome Sanger Institute Scientific Operations: Sequencing Operations are listed here: https://doi.org/10.5281/zenodo.10043364.

Members of the Wellcome Sanger Institute Tree of Life Core Informatics team are listed here: https://doi.org/10.5281/zenodo.10066637.

Members of the Tree of Life Core Informatics collective are listed here: https://doi.org/10.5281/zenodo.5013541.

Members of the Darwin Tree of Life Consortium are listed here: https://doi.org/10.5281/zenodo.4783558.
