# Supplementary material for: The genome sequence of the Fig-leaf Skeletoniser, Choreutis nemorana (Hübner, [1799])
Source: Wellcome Open Res. 2024 May 15;9:268. [Version 1] doi: 10.12688/wellcomeopenres.21624.1 (PMC11406129; doi:10.12688/wellcomeopenres.21624.1)
Supplement: Peer review history

# Reviewer report: Reviewer response for version 1

**DOI:** 10.21956/wellcomeopenres.23896.r94631

**Published:** 2024-09-16

**Authors:** Ann-Marie Waldvogel

**Affiliations:** 1 University of Cologne, Cologne, Germany

This genome report describes and summarizes the details of a de novo genome assembly of the moth species *Choreutis nemorana*. The value and relevance of novel de novo assembly should not be questioned in general and this particular genomes will contribute to future research on insect/lepidopteran phylogenies and functional studies.

 I highly recommend to include a nicer and more informative picture that shows the adult moth. Especially, since it has been described in its adult morphology in the background section. The provided photo is a visual voucher of the sequenced individual but not really representative for the species.

 The assembly pipeline applies state-of-the-art approaches and the results are documented according to standard. It can be recommended to use Braker3 for future annotations, as here the pipeline used Barker2.

Are sufficient details of methods and materials provided to allow replication by others?

Yes

Is the rationale for creating the dataset(s) clearly described?

Yes

Are the datasets clearly presented in a useable and accessible format?

Yes

Are the protocols appropriate and is the work technically sound?

Yes

Reviewer Expertise:

biodiversity genomics, ecological genomics, population genomics

I confirm that I have read this submission and believe that I have an appropriate level of expertise to confirm that it is of an acceptable scientific standard.

# Reviewer report: Reviewer response for version 1

**DOI:** 10.21956/wellcomeopenres.23896.r94628

**Published:** 2024-09-07

**Authors:** Sivasankaran Kuppusamy

**Affiliations:** 1 Entomology Research Institute, Loyola College, Chennai, India

The genome of the Fig-leaf Skeletonizer, *Choreutis nemorana *(Hubner, [1799]), has been sequenced by authors. 300.2 megabases is the total size of the assembled genome. Using the genome annotations, the authors have discovered 15,351 protein-coding genes and 15,541 gene transcripts. They have used proper procedure for nucleic acid extraction and appropriate software for assembly and annotations.

**Comments on the manuscript:**

- In the Background fifth paragraph third line “ *and”* is in italics. It can be changed.  
- In the Genome sequence report the first sentence can be written as “The genome was sequenced from a male caterpillar of *C. nemorana*……..”
- In the Methods the genus and species names were merged. The genus name and species can be separated. The sentence can be written as “A *C. nemorana* caterpillar (specimen ID NHMUK013696811, ToLID ilChoNemo1) was collected from Kensington….

Above all, I confirm that the manuscript meets the necessary scientific standard and is suitable for Indexed".

Are sufficient details of methods and materials provided to allow replication by others?

Yes

Is the rationale for creating the dataset(s) clearly described?

Yes

Are the datasets clearly presented in a useable and accessible format?

Yes

Are the protocols appropriate and is the work technically sound?

Yes

Reviewer Expertise:

Phylogenetic analysis of Noctuoidea moths using NGS techniques

I confirm that I have read this submission and believe that I have an appropriate level of expertise to confirm that it is of an acceptable scientific standard.
